# Precision prognosis of colorectal cancer: a multi-tiered model integrating microsatellite instability genes and clinical parameters

**DOI:** 10.3389/fonc.2024.1396726

**Published:** 2024-07-11

**Authors:** Yonghong Wang, Ke Liu, Wanbin He, Jie Dan, Mingjie Zhu, Lei Chen, Wenjie Zhou, Ming Li, Jiangpeng Li

**Affiliations:** Department of Gastrointestinal Surgery, The People's Hospital of Leshan, Leshan, China

**Keywords:** enhancing CRC survival prediction colorectal cancer, prognostic model, microsatellite instability, survival, prediction

## Abstract

**Background:**

Prognostic assessment for colorectal cancer (CRC) displays substantial heterogeneity, as reliance solely on traditional TNM staging falls short of achieving precise individualized predictions. The integration of diverse biological information sources holds the potential to enhance prognostic accuracy.

**Objective:**

To establish a comprehensive multi-tiered precision prognostic evaluation system for CRC by amalgamating gene expression profiles, clinical characteristics, and tumor microsatellite instability (MSI) status in CRC patients.

**Methods:**

We integrated genomic data, clinical information, and survival follow-up data from 483 CRC patients obtained from The Cancer Genome Atlas (TCGA) and Gene Expression Omnibus (GEO) databases. MSI-related gene modules were identified using differential expression analysis and Weighted Gene Co-expression Network Analysis (WGCNA). Three prognostic models were constructed: MSI-Related Gene Prognostic Model (Model I), Clinical Prognostic Model (Model II), and Integrated Multi-Layered Prognostic Model (Model III) by combining clinical features. Model performance was assessed and compared using Receiver Operating Characteristic (ROC) curves, Kaplan-Meier analysis, and other methods.

**Results:**

Six MSI-related genes were selected for constructing Model I (*AUC* = 0.724); Model II used two clinical features (*AUC* = 0.684). Compared to individual models, the integrated Model III exhibited superior performance (*AUC* = 0.825) and demonstrated good stability in an independent dataset (*AUC* = 0.767).

**Conclusion:**

This study successfully developed and validated a comprehensive multi-tiered precision prognostic assessment model for CRC, providing an effective tool for personalized medical management of CRC.

## 1 Introduction

Colorectal cancer (CRC) is a malignant neoplasm that arises from the mucosal epithelium of the colon or rectum, constituting a significant global public health concern (1). Global statistics from the International Agency for Research on Cancer indicate that CRC has emerged as the second leading cause of cancer-related mortality, with over 1.9 million new cases being reported in 2020. Projections suggest that by 2040, there will be a staggering 3.2 million new cases of CRC worldwide, imposing a significant burden on both patients and society (2).

The pathogenesis of CRC is notably complex, often encompassing multiple stages of genetic and epigenetic changes within colonic epithelial cells (3). Notably, the inactivation of tumor suppressor genes, including *APC*, *KRAS*, *TP53*, and the activation of DNA mismatch repair genes are regarded as pivotal mechanisms in the formation of CRC (4). These traditional markers have been pivotal in understanding CRC’s development but offer limited prognostic precision due to their common alterations across many patients with diverse outcomes. Furthermore, factors such as age, obesity, smoking, and alcohol misuse play critical roles in the increased incidence of CRC (5–7).

Despite advancements in CRC screening methods and the introduction of innovative therapies providing hope to patients, the prognostic assessment of CRC still carries a degree of uncertainty due to the intricacies of individual variations. While the widely employed TNM staging system in clinical practice categorizes the risk of CRC, its predictive performance for patients’ prognosis remains variable. Research has indicated that even among patients within the same TNM staging, differences in the 5-year survival rate can be as substantial as 30% (8). This primarily arises from the complexity of individual physiological conditions, making reliance solely on TNM staging insufficient for precise prognostication. Consequently, researchers have endeavored to establish more precise and comprehensive predictive models to facilitate individualized assessments. Nevertheless, prior models have predominantly depended on individual clinical markers or specific biomolecules (9–11). For example, certain studies have applied molecular biology analyses and biomarker identification techniques to evaluate distinct genes, assessing their influence on the survival and prognosis of CRC (12, 13). However, these studies have not comprehensively accounted for the intricacies of tumor biology. Concurrently, some investigations have narrowed their scope to clinical indicators, overlooking alterations at the molecular level (14). These studies have not comprehensively addressed the multi-tiered biological characteristics of the disease, consequently restricting their accuracy and applicability.

In light of the limitations of the aforementioned models, the pursuit of markers that reflect the intricacies of tumor genomics has emerged as a primary focus of research. We have identified Microsatellite Instability (MSI) as a characteristic indicative of DNA repair deficiencies. This manifests as instability in DNA microsatellite repeat sequences, leading to DNA mismatches and the accumulation of mutations within tumor cells. Presently, MSI has been comprehensively investigated and presents distinctive biological and clinical attributes in numerous cancer types (15, 16). Unlike the broad variations involving traditional markers such as *APC* and *TP53*, MSI provides a more direct indication of tumor behavior, particularly in terms of response to certain therapies and survival rates, making it an invaluable tool for enhancing personalized medicine. Through the examination of genes and biological processes linked to MSI, we can attain a more profound understanding of the biological features of CRC.

**Figure 1:**
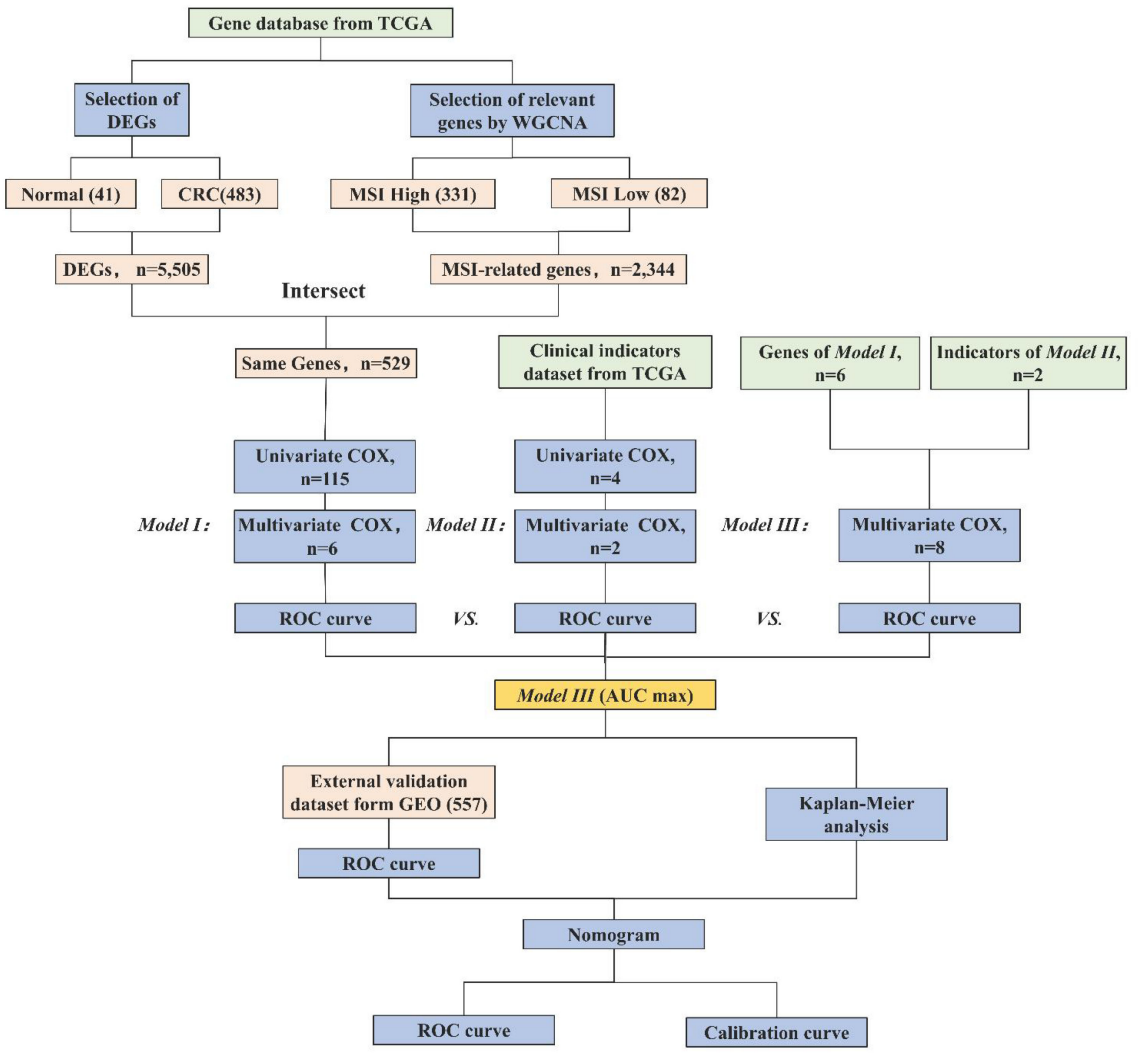
Research workflow of construction of the CRC prognostic model. Abbreviations: TCGA (The Cancer Genome Atlas), DEGs (Differentially Expressed Genes), WGCNA (Weighted Gene Co-expression Network Analysis), MSI (Microsatellite Instability), ROC (Receiver Operating Characteristic), AUC (Area Under the Curve), and GEO (Gene Expression Omnibus). Model I (MSI-Related Gene Prognostic Model), Model II (Clinical Prognostic Model), Model III (Integrated Multi-Layered Prognostic Model).

Therefore, in this study, we employ MSI-related genes to build a predictive model, integrating them with clinical parameters to augment the precision and comprehensiveness of CRC prognostic prediction. We assess the effectiveness of gene models, clinical models, and integrated models. Additionally, we employ visualization tools to augment the clinical applicability of our models. The research process is shown in **Figure 1**. This innovative approach not only holds the potential to enhance the accuracy of treatment and survival outcomes for CRC patients but also to deepen our comprehension of its prognostic mechanisms.

## 2 Methods

### 2.1 Data acquisition and preprocessing

The Coad dataset from the Cancer Genome Atlas (TCGA) database was employed, encompassing gene expression profiles, clinical characteristics, and prognosis information of 483 CRC patients. Data from TCGA were accessed between September 18, 2023, and September 26, 2023, ensuring the use of the most current data available at the time of analysis. A series of preprocessing steps were applied to the data, including the removal of duplicate samples, exclusion of genes and clinical features with missing proportions exceeding 30%, and imputation of remaining missing data using mean values. Ultimately, we obtained an integrated dataset comprising 19,937 genes and 9 clinical features. To facilitate external validation, the GSE39582 dataset from the Gene Expression Omnibus (GEO) database, which includes 557 samples, was also utilized.

### 2.2 Construction of model I

#### 2.2.1 Differentially expressed genes selection

The “limma” package in R was employed to calculate DEGs between cancerous and normal tissues. Our selection criteria included a fold change > 2 and an adjusted *P*-value < 0.05. This threshold was selected based on established practices in oncological research (17), where such a level of expression change is considered significant enough to potentially contribute to cancer pathogenesis and progression. It allows for the discrimination of genes most likely to have substantial biological effects, focusing on changes that are more likely to influence disease outcomes and patient prognosis. Choosing a *P*-value < 0.05 helps balance sensitivity and specificity, effectively reducing the risk of Type I errors while still capturing genes with potentially significant biological differences (18). This approach not only aligns with standard practices in oncological research but is particularly suited to exploratory studies aimed at mapping comprehensive gene expression landscapes and identifying novel targets for further validation.

#### 2.2.2 Weighted gene co-expression network analysis

To identify gene modules and core genes associated with MSI, the WGCNA algorithm was employed. Initially, the gene expression matrix was transformed into a matrix containing pairwise mRNA similarity among genes. Subsequently, this was converted into an adjacency matrix using Pearson correlation coefficients. The construction of a scale-free network ensured the adjacency matrix adhered to scale-free topological criteria. Topological overlap matrices (TOM) and dissimilarity TOM (diss TOM) were then created for further analysis. Lastly, dynamic tree cutting was utilized to identify modules, with the minimum module size set at 20 to obtain highly similar modules, combined with thresholds for each dataset.

#### 2.2.3 Univariate Cox regression analysis

In this step, an intersection was taken between DEGs and MSI-associated genes identified through WGCNA, followed by selection through univariate Cox regression analysis. Genes with *P*-values below 0.05 were included in the final gene list.

#### 2.2.4 Construction of multivariate COX model

To construct the gene prognosis model, a forward stepwise regression method was employed, which retained genes associated with independent prognosis and formed gene weights.

### 2.3 Construction of model II

#### 2.3.1 Univariate survival analysis

Univariate Cox regression analysis was conducted on clinical features, with features having *P* < 0.05 selected.

#### 2.3.2 Multivariate COX model

Utilizing forward stepwise regression, a multivariate Cox model was built, preserving clinical features associated with independent prognosis, thereby forming the clinical feature prognosis assessment model.

### 2.4 Construction of model III

In this step, MSI-related genes selected from Model I were combined with the clinical model constructed in Model II, creating a comprehensive multi-tiered prognosis model for CRC. Through the integration of genes and clinical indicators, a multivariate Cox regression analysis was utilized to construct a multi-tiered CRC prognosis model.

### 2.5 Model comparison

To compare the performance of Model I, Model II, and Model III prognosis models, Receiver Operating Characteristic (ROC) curve analysis was conducted, and area Under the Curve (AUC) values were calculated. Ultimately, the model with the highest AUC value was chosen for external validation to assess its generalizability and stability.

### 2.6 Survival analysis

Survival analysis was conducted on factors used to build the models, Kaplan-Meier survival curves were plotted, and inter-group comparisons were performed using the Log-rank test. All survival curves were generated using R 4.3.2 software.

### 2.7 Model visualization

Nomogram was constructed for the optimal-performing model to visually demonstrate its performance. Furthermore, ROC curves for 1-year, 2-year, and 3-year predictions were plotted to evaluate the model’s predictive accuracy, and calibration curves were used to validate the model’s calibration.

### 2.8 Statistical analysis

Statistical analysis of the experimental data in this study was conducted using R software version 4.3.1. Kaplan-Meier survival curves were compared between groups using the Log-rank test, with statistical significance set at *P*-value < 0.05.

### 2.9 Data availability statement

The data employed in this study are accessible through publicly available repositories. The gene expression data and clinical records for patients with CRC were sourced from TCGA database (https://www.cancer.gov/tcga). Specifically, the dataset employed is denoted as “Coad,” encompassing comprehensive gene expression profiles and associated clinical characteristics. For external validation, we made use of the GEO database (https://www.ncbi.nlm.nih.gov/geo/), with the selected dataset identified as “GSE39582.” All datasets utilized in this research are openly accessible for retrieval, subject to the terms of use and data access policies stipulated by the respective databases. Should any further details or inquiries pertaining to data availability be required, they may be directed to the corresponding author.

## 3 Results

### 3.1 WGCNA modules with MSI and identification of DEGs

**Figure 2:**
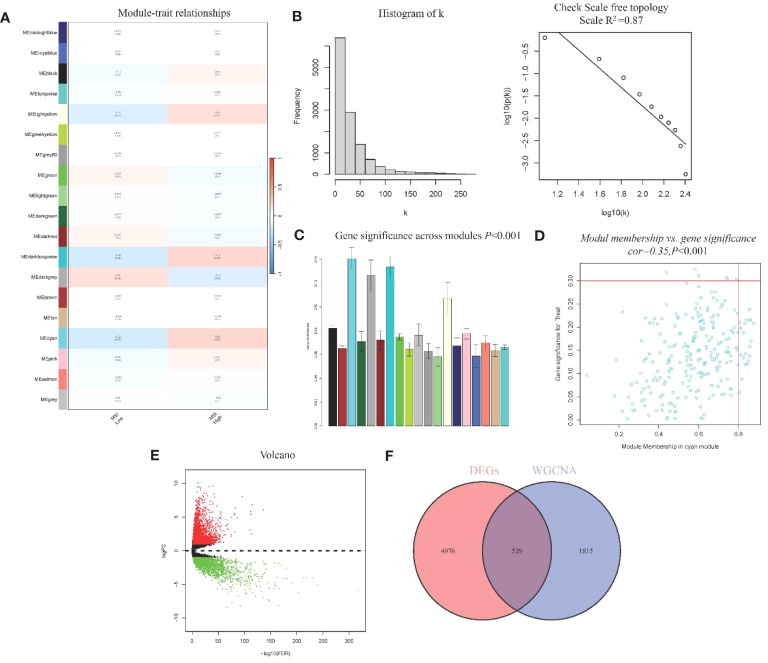
WGCNA module analysis and Identification of DEGs **(A)** Heatmap of Association of Gene Modules with MRI in CRC; **(B)**. Log-log plot of whole-network connectivity distribution; **(C)** Barplot of mean gene significance across modules; **(D)** Scatter plots of the blue module eigengene value showing correlation with MSI status; **(E)** Volcano plot of DEGs; **(F)** Venn diagram of overlap between DEGs and module genes.

WGCNA analysis identified 19 gene modules and their associations with MSI in CRC (**Figure 2A**). We excluded obvious outlier samples by setting appropriate thresholds. A soft threshold of 20 was applied, resulting in high average connectivity (*R* (2) = 0.87, **Figure 2B**). Modules were associated with clinical features based on module eigengenes’ correlation with clinical symptoms, and a total of 12,405 genes were screened out (**Figure 2C**). The blue module contained 2,344 genes, which were strongly positive correlation with MSI (*r* = 0.23, *P* = 0.02) and negatively correlation with low MSI (*r* = -0.23, *P* = 0.02), which had clinical significance (**Figure 2D**). A total of 5,505 DEGs were identified, consisting of 3,175 upregulated genes and 2,330 downregulated genes. A volcano plot visually represented the differential expression patterns (**Figure 2E**).

### 3.2 Model I: MSI-related gene prognostic model

**Figure 3:**
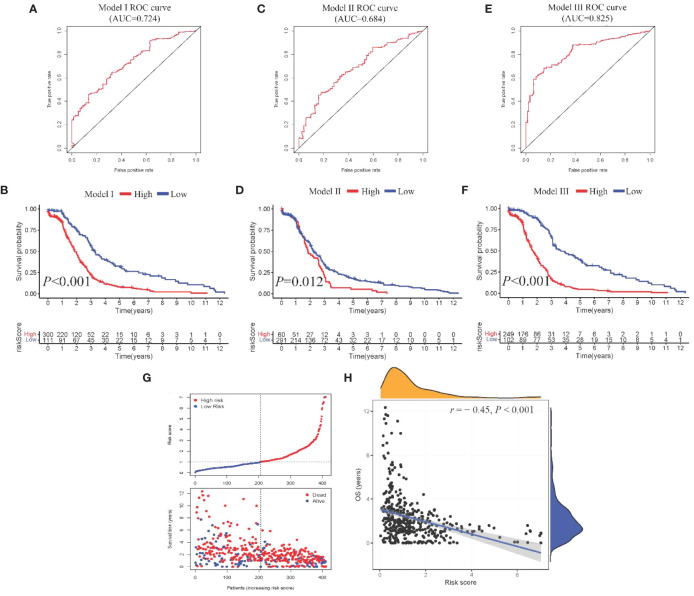
Construction and comparison of prognostic models. **(A, B)**. ROC curve and Kaplan-Meier curves of Model I; **(C, D)** ROC curve and Kaplan-Meier curves of Model II; **(E, F)**. ROC curve and Kaplan-Meier curves of Model III; **(G)** Scatter plot showing risk score stratification and mortality for Model III; **(H)** Scatter plot depicting the correlation between Model III risk score and survival time.

From the DEGs and modules derived from WGCNA, 529 genes were selected (**Figure 2F**). Subsequently, univariate Cox regression analysis was conducted to identify genes associated with the prognosis of CRC patients. This analysis revealed that 115 genes had significant prognostic value (**Supplementary Table 1**). In the multivariate Cox model, six independent prognostic-related genes were identified, namely *GNL3*, *VSIR*, *LY86*, *ARHGAP25*, *DERL3*, and *JAML* (**Table 1**). LASSO regression further validated these genes (**Supplementary Figure 1**). Further analysis demonstrated that the model had an AUC of 0.724 (**Figure 3A**), indicating robust predictive performance. Additionally, Kaplan-Meier survival curves showed a significant difference between high-risk and low-risk patients (*P* < 0.001, **Figure 3B**).

**Table 1:** Genes Associated with CRC Prognosis.

Genes	HR	HR.95L	HR.95H	*P-*value
*GNL3*	0.346	0.219	0.546	<0.001
*VSIR*	0.440	0.275	0.704	<0.001
*LY86*	0.356	0.199	0.638	<0.001
*ARHGAP25*	9.243	2.764	30.903	<0.001
*DERL3*	0.315	0.178	0.557	<0.001
*JAML*	5.510	2.238	13.558	<0.001

### 3.3 Model II: clinical prognostic model

Univariate Cox regression analysis identified four features: T, N, M, and stage (**Supplementary Table 2**). The multivariate Cox regression model included two independent prognostic factors: M and stage (**Table 2**). The model’s AUC was 0.684 (**Figure 3C**), demonstrating reasonable predictive performance. Kaplan-Meier survival curves also displayed a significant separation between high-risk and low-risk patients (*P* = 0.012, **Figure 3D**).

**Table 2:** Clinical Features Associated with CRC.

Clinical Features	HR	HR.95L	HR.95H	*P-*value
M	4.014	2.721	5.921	<0.001
Stage	2.768	1.306	5.869	<0.001

### 3.4 Model III: integrated multi-layered prognostic model

Integration of Model I and Model II resulted in a comprehensive CRC prognosis model comprising six MSI-related genes (*GNL3*, *VSIR*, *LY86*, *ARHGAP25*, *DERL3*, *JAML*) and two clinical features (M and stage). The AUC for this comprehensive model reached 0.825 (**Figure 3E**), indicating outstanding predictive performance. Kaplan-Meier survival curves further demonstrated a significant difference between high-risk and low-risk patients (*P* < 0.001, **Figure 3F**).

### 3.5 Models comparison and external validation

**Figure 4:**
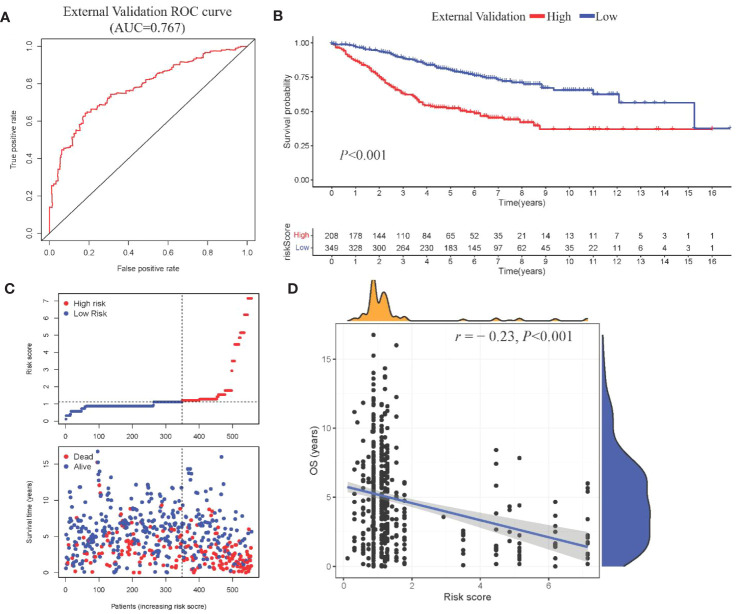
External validation of Model III **(A)** ROC curve in validation cohort; **(B)** Kaplan-Meier curve showing survival stratification in validation cohort; **(C)**Scatter plot depicting risk score stratification and mortality in the external validation cohort; **(D)** Scatter plot illustrating the correlation between risk score and survival time in the external validation cohort.

**Figure 5:**
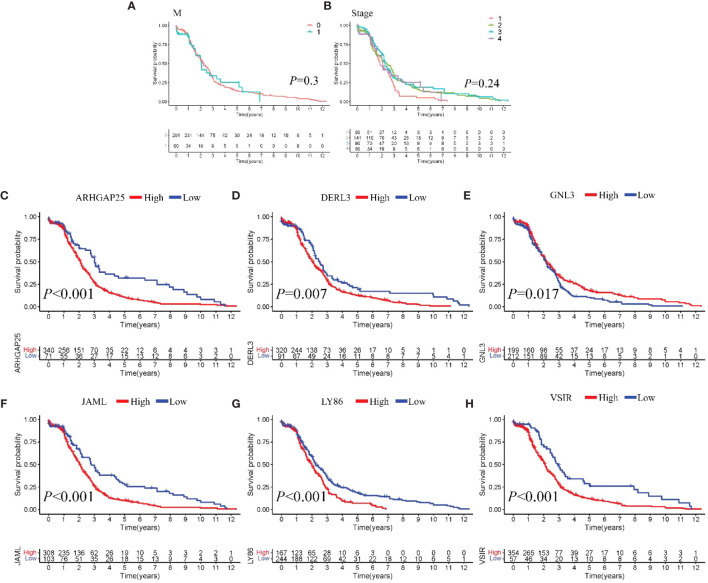
Survival analysis **(A-H).** Kaplan-Meier curves of individual factors in validation cohort.

Model III exhibited the highest AUC value compared to Model I and Model II (0.825 *vs.* 0.724 *vs.* 0.684). Risk factor analysis indicated a positive correlation between risk scores and patient mortality (**Figure 3G**). Moreover, higher risk scores were associated with shorter patient survival times (*P* < 0.001, **Figure 3H**). External validation confirmed the stability of Model III across different datasets (AUC = 0.767, **Figure 4A**). Kaplan-Meier curves in the external validation set further demonstrated the model’s ability to effectively distinguish high-risk and low-risk patients (*P* < 0.001, **Figure 4B**). Risk factor analysis in the external validation set revealed a positive correlation between risk scores and patient mortality (**Figure 4C**). Furthermore, higher risk scores were associated with shorter patient survival times (*P* < 0.001, **Figure 4D**). Kaplan-Meier curves for each factor in the external validation set reaffirmed these findings (**Figures 5A–H**), emphasizing the effectiveness of Model III. To further validate the relevance of specific genes included in our analysis, we consulted the Human Protein Atlas, where survival plots for the six genes show significant prognostic associations (**Supplementary Figure 2**).

### 3.6 Visualization of model III

**Figure 6:**
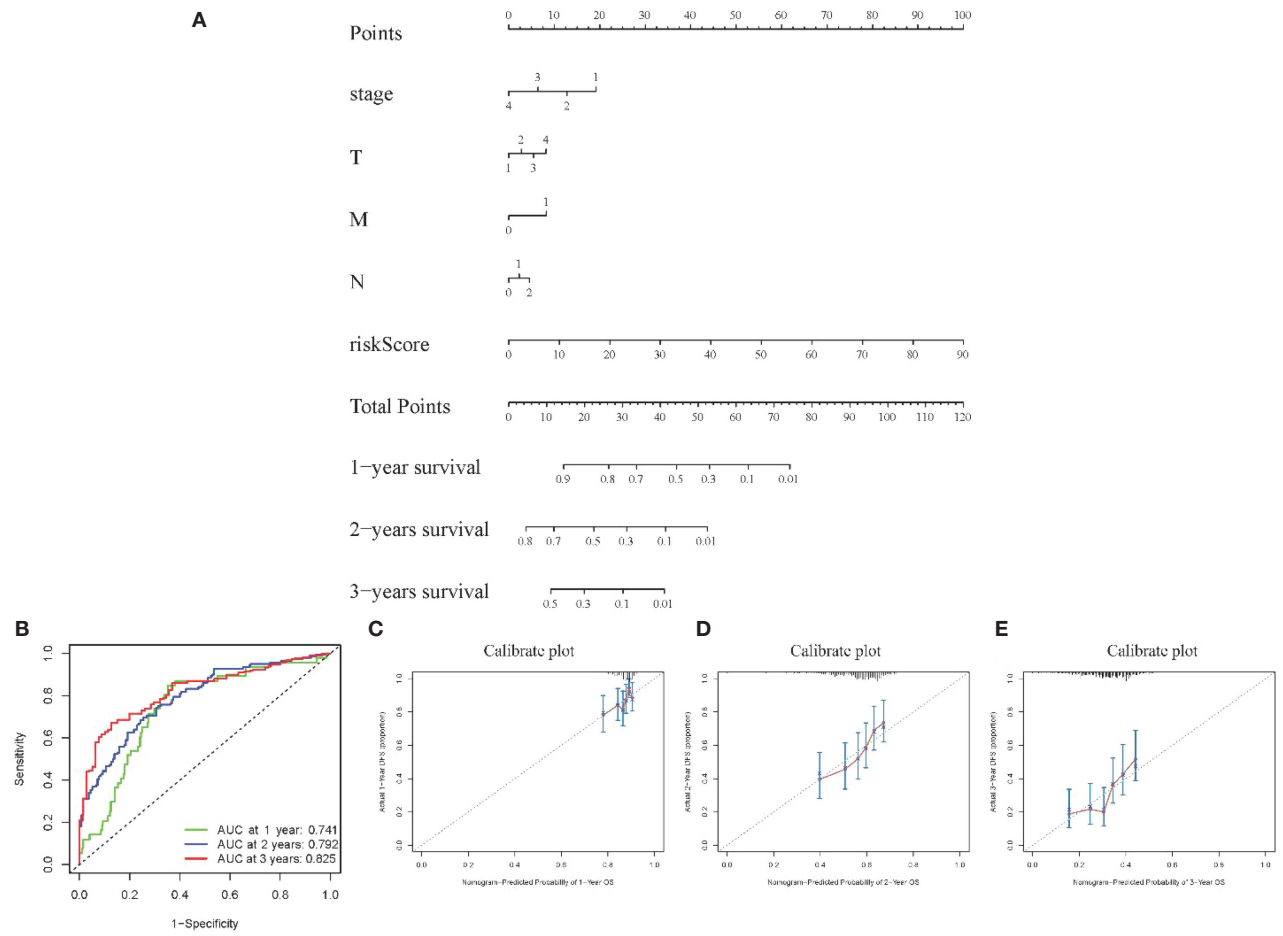
Visualization of Model III **(A)** Nomogram showing 1–3-year survival prediction; **(B)** ROC curves showing discrimination ability; **(C-E)** Calibration curves assessing prediction accuracy over 1-3 years.

Visualization nomogram presented survival rate predictions at 1 year, 2 years, and 3 years (**Figure 6A**). ROC curves for 1 year, 2 years, and 3 years were included in the nomogram, all with AUC values exceeding 0.7 (**Figure 6B**), indicating good capability in differentiating patient survival rates. Additionally, calibration curves at 1 year, 2 years, and 3 years were presented in **Figures 6C–E**, respectively. These calibration curves demonstrated the consistency between the model’s predicted risk and observed risk, highlighting the model’s good calibration and enhancing its reliability and practicality in clinical applications.

## 4 Discussion

In the realm of CRC prognosis research, we have effectively crafted a sophisticated, multi-tiered comprehensive model that seamlessly integrates gene expression, clinical characteristics, and MSI status. This achievement introduces a novel instrument for tailored treatment strategies. Through the amalgamation of these pivotal elements, we have not only enhanced the precision of CRC prognosis predictions but also opened up an avenue for healthcare practitioners and patients to delve deeper into tumor intricacies and identify personalized treatment trajectories.

An eminent innovation in this investigation is the assimilation of MSI status as a prominent prognostic indicator within the model. Although MSI is a recognized prognostic biomarker for CRC (19), our research bestows a new dimension upon its integration and application. Firstly, the inclusion of MSI status in our model inherently augments CRC prognosis predictions. The well-established association between elevated MSI levels in CRC and improved prognosis bolsters the predictive accuracy of the model. Secondly, MSI can be viewed as an intermediate milestone connecting downstream molecular events, thereby aiding in the prediction of ultimate survival outcomes (20). Through WGCNA analysis, we have unearthed a strong correlation between MSI status and specific gene modules, further substantiating the scientific soundness of our model strategy. This underscores not only the significance of MSI in CRC biology but also furnishes a robust foundation for future explorations into MSI mechanisms (21, 22).

Our study has formulated a comprehensive, multi-layered model, which following rigorous validation. In comparison to single-feature models, this multi-layered model incorporates a medley of features, encompassing gene expression and clinical characteristics. This results in heightened effectiveness and consistent performance, even when applied to external datasets. This underlines the notion that single biomarkers or clinical features inadequately capture the intricacies of CRC. However, our model adeptly amalgamates information from various levels, thus bridging the divide between genomics and clinical data. This integrative approach expands the scope of our predictive system, spanning multiple strata from molecular to individual, and consequently, it significantly enhances predictive accuracy, thereby providing robust support for future clinical applications.

The study has identified six gene expression-related biomarkers: *GNL3*, *VSIR*, *LY86*, *ARHGAP25*, *DERL3*, and J*AML*—linked to key biological processes such as cytokine metabolism and oxidative stress, which are vital for regulating cell proliferation, apoptosis, and invasion in CRC. Additionally, the study suggests that MSI may influence these biomarkers, potentially intensifying cancer progression in MSI-high CRC cases. *GNL3* encodes nucleolin, a critical regulator of cell proliferation. It maintains normal cell growth by modulating the p53 pathway and facilitating DNA damage repair. Prior research (23) has established that reduced expression of *GNL3* may compromise DNA replication and genomic stability, contributing to CRC progression. In MSI-high CRC, the disruption of nucleolar processes and DNA repair by MSI-induced mutations may further exacerbate genomic instability, underlining the crucial role of *GNL3* in maintaining genomic integrity. *VSIR* encodes a receptor in the VEGF pathway, promoting angiogenesis in tumors. Elevated expression of VSIR activates downstream pathways such as MAPK and AKT, enhancing the potential for CRC cell invasion and metastasis (24). This effect might be amplified in MSI-high CRC, where genetic alterations could heighten the responsiveness of CRC cells to VEGF signaling, leading to more aggressive tumor phenotypes. *LY86* participates in Toll-like receptor signaling and tumor microenvironment regulation. Dysregulated *LY86* expression can lead to abnormal inflammatory responses and diminished immune surveillance, promoting CRC development (25). In MSI-high tumors, altered *LY86*-mediated signaling could contribute to an immunosuppressive microenvironment that favors tumor escape. *ARHGAP25*, governing cell cytoskeleton organization and movement, exhibits increased expression in CRC cells, correlating with enhanced migration and invasion capabilities (26). The role of *ARHGAP25* might be particularly pronounced in MSI-high CRC, potentially leading to increased tumor invasiveness and metastatic capacity due to heightened mutation rates affecting cell motility. *DERL3* mediates the endoplasmic reticulum stress response. Aberrant function in *DERL3* can lead to unfolded protein accumulation and apoptosis suppression, enhancing CRC cell survival and progression (27). In the context of MSI-high CRC, exacerbated protein folding disorders could intensify reliance on *DERL3* for maintaining cell survival under stress conditions. *JAML*, an adhesion molecule on myeloid cells, interacts with JAM-C, promoting tumor cell extravasation and metastasis. Elevated expression of *JAML* indicates advanced disease progression and poorer prognosis (28). In MSI-high CRC, enhanced interaction with JAM-C could increase metastatic potential and worsen prognosis due to altered adhesion dynamics. These speculative interactions between MSI status and gene expression highlight potential targets for future research and therapeutic intervention, suggesting that MSI might significantly influence the pathophysiological landscape of CRC through these key molecular pathways.

Furthermore, the study emphasizes the significance of these two critical clinical characteristics: Stage and M. They have consistently been recognized as fundamental parameters for evaluating CRC patient prognosis according to guidelines (29). Stage reflects not only the tumor’s size and degree of infiltration but also serves as a crucial foundation for assessing the patient’s condition’s severity (30). Advancing stage typically indicates tumor spread to surrounding tissues or lymph nodes, consequently heightening treatment complexity and the risk of a worse prognosis. And M, indicating the presence of metastatic lesions in distant organs. This factor directly influences the selection of treatment regimens and the assessment of prognosis (31). When the tumor disseminates to distant organs, patients frequently face more complex and aggressive treatments, leading to a poorer prognosis. The study’s findings reaffirm the close connection between Stage and M in the context of CRC prognosis, emphasizing their crucial roles in CRC prognosis assessment.

In addition to our multi-layered comprehensive model, we have developed an intuitive visualization tool known as a nomogram. The development of this nomogram is aimed at enhancing clinicians’ ability to assess the prognosis risk of patients with CRC more effectively, thereby facilitating the formulation of personalized treatment strategies (32, 33). The utilization of the nomogram is straightforward, as clinicians can input patients’ gene expression data and clinical features into the chart, resulting in the generation of individualized prognosis risk scores. The chart transparently presents survival rate predictions for CRC patients at 1 year, 2 years, and 3 years. This visualization method empowers clinicians to swiftly distinguish between high-risk and low-risk patients, thereby facilitating the development of more tailored treatment plans.

Our prognostication model has demonstrated potential in enhancing the accuracy of predictions in CRC. However, this study, despite its significant innovations, does exhibit certain limitations. Firstly, it is important to note that the model has not yet been tested in clinical settings. We acknowledge that field-testing is crucial to confirm the model’s applicability and reliability in real-world scenarios. Plans are underway to conduct such validation, which we believe will be pivotal in establishing the model’s true effectiveness. This crucial step will help bridge the gap between theoretical research and practical clinical application, ensuring that our model can be reliably used in the management of CRC. Secondly, the precise mechanisms underlying the biomarkers included in the model remain unelucidated, necessitating further experimental research to uncover their modes of action. Lastly, despite the integration of multi-layered information, it is conceivable that other unconsidered biological factors may exist. Future research endeavors should aim to refine the model by incorporating these factors.

In summary, this study successfully constructed a multi-layered prognostic model for CRC that seamlessly integrates gene expression and clinical features. This model not only enhances predictive accuracy but also provides robust support for future clinical practice through the use of a nomogram as a visualization tool.

## Data availability statement

The original contributions presented in the study are included in the article/**Supplementary Material.** Further inquiries can be directed to the corresponding author.

## Author contributions

YW: Conceptualization, Writing – original draft, Writing – review & editing. KL: Data curation, Methodology, Writing – review & editing. WH: Formal Analysis, Validation, Writing – review & editing. JD: Writing – review & editing. MZ: Writing – review & editing, Data curation. LC: Data curation, Methodology, Writing – review & editing. WZ: Writing – review & editing. ML: Writing – review & editing. JL: Writing – review & editing.
