# Supplementary material for: Precision prognosis of colorectal cancer: a multi-tiered model integrating microsatellite instability genes and clinical parameters
Source: Front Oncol. 2024 Jul 11;14:1396726. doi: 10.3389/fonc.2024.1396726 (PMC11269184; doi:10.3389/fonc.2024.1396726)

Supplementary Table 1 Genes Associated with CRC Prognosis in Univariate Cox Analysis

| gene | HR | z | pvalue |
| --- | --- | --- | --- |
| MPEG1 | 1.271488 | 4.279977 | 1.87E-05 |
| AC136428.1 | 1.384992 | 4.239742 | 2.24E-05 |
| SLAMF7 | 1.265299 | 4.149172 | 3.34E-05 |
| MZB1 | 1.183367 | 4.125481 | 3.70E-05 |
| EVI2B | 1.274425 | 3.838722 | 0.000124 |
| GPR34 | 1.304553 | 3.775013 | 0.00016 |
| IRF4 | 1.400034 | 3.697483 | 0.000218 |
| PPP1R16B | 1.391091 | 3.682151 | 0.000231 |
| BHLHA15 | 1.394732 | 3.650753 | 0.000261 |
| P2RY13 | 1.362698 | 3.544856 | 0.000393 |
| CMKLR1 | 1.321217 | 3.525643 | 0.000422 |
| IGLL5 | 1.310951 | 3.494388 | 0.000475 |
| FGL2 | 1.247175 | 3.486007 | 0.00049 |
| SASH3 | 1.254122 | 3.485636 | 0.000491 |
| TNFRSF17 | 1.228419 | 3.454658 | 0.000551 |
| CD27 | 1.292376 | 3.424771 | 0.000615 |
| CSF1R | 1.197864 | 3.421211 | 0.000623 |
| GIMAP6 | 1.283265 | 3.409441 | 0.000651 |
| JAML | 1.418967 | 3.401852 | 0.000669 |
| EBI3 | 1.365793 | 3.348378 | 0.000813 |
| ADA2 | 1.223221 | 3.341338 | 0.000834 |
| CLEC10A | 1.274161 | 3.330827 | 0.000866 |
| GPR171 | 1.442239 | 3.325721 | 0.000882 |
| ZCCHC24 | 1.240022 | 3.318927 | 0.000904 |
| IKZF1 | 1.433668 | 3.281845 | 0.001031 |
| TMEM273 | 1.358714 | 3.254976 | 0.001134 |
| RCSD1 | 1.410595 | 3.245883 | 0.001171 |
| TAGAP | 1.349282 | 3.23216 | 0.001229 |
| MS4A6A | 1.21982 | 3.20014 | 0.001374 |
| POU2AF1 | 1.236615 | 3.122969 | 0.00179 |
| IL10RA | 1.265476 | 3.120468 | 0.001806 |
| CHI3L2 | 1.392233 | 3.099501 | 0.001938 |
| PRKCB | 1.452614 | 3.095004 | 0.001968 |
| P2RY10 | 1.441592 | 3.08714 | 0.002021 |
| ITGAL | 1.323838 | 3.082201 | 0.002055 |
| HCLS1 | 1.224387 | 3.076471 | 0.002095 |
| CD209 | 1.332593 | 3.057082 | 0.002235 |
| TBC1D9 | 1.285534 | 3.045425 | 0.002324 |
| EPB41L3 | 1.26423 | 3.04306 | 0.002342 |
| NCKAP1L | 1.272038 | 3.022089 | 0.00251 |
| PPP2R2C | 0.755101 | -2.98055 | 0.002877 |
| BTK | 1.371343 | 2.953087 | 0.003146 |
| APBB1IP | 1.270979 | 2.933118 | 0.003356 |
| VAMP2 | 1.386771 | 2.907816 | 0.00364 |
| DOCK2 | 1.315917 | 2.902569 | 0.003701 |
| CD48 | 1.257918 | 2.89097 | 0.003841 |
| RAB27A | 1.317915 | 2.890757 | 0.003843 |
| MAP4K1 | 1.361581 | 2.887802 | 0.003879 |
| MS4A7 | 1.214473 | 2.871271 | 0.004088 |
| KLRB1 | 1.285104 | 2.866154 | 0.004155 |
| EML1 | 1.385263 | 2.862333 | 0.004205 |
| GIMAP4 | 1.197994 | 2.837613 | 0.004545 |
| ARHGAP25 | 1.352502 | 2.809274 | 0.004965 |
| IRAG2 | 1.457646 | 2.806821 | 0.005003 |
| ITGB7 | 1.303867 | 2.802671 | 0.005068 |
| CD163 | 1.131298 | 2.791491 | 0.005247 |
| CD3E | 1.190056 | 2.779077 | 0.005451 |
| C1QB | 1.124547 | 2.76599 | 0.005675 |
| PLPP3 | 1.302685 | 2.7349 | 0.00624 |
| SLA2 | 1.370592 | 2.734366 | 0.00625 |
| CCR2 | 1.32563 | 2.707526 | 0.006779 |
| VSIR | 1.249948 | 2.70583 | 0.006813 |
| PLA2G2D | 1.237757 | 2.704059 | 0.00685 |
| MTM1 | 1.343509 | 2.66168 | 0.007775 |
| CD96 | 1.330486 | 2.661246 | 0.007785 |
| CD8A | 1.16974 | 2.649021 | 0.008073 |
| ACSL4 | 1.205369 | 2.646783 | 0.008126 |
| TENT5C | 1.182382 | 2.637034 | 0.008363 |
| ERICH2 | 0.831664 | -2.61258 | 0.008986 |
| CT83 | 1.196099 | 2.609841 | 0.009058 |
| FADS3 | 1.301414 | 2.60799 | 0.009108 |
| GAB1 | 1.442186 | 2.605443 | 0.009176 |
| MS4A4A | 1.168576 | 2.504602 | 0.012259 |
| B2M | 1.198202 | 2.491139 | 0.012733 |
| ADAM28 | 1.286622 | 2.491015 | 0.012738 |
| RRP1 | 0.758279 | -2.48377 | 0.013 |
| WNT5A | 1.20061 | 2.471884 | 0.01344 |
| PLEKHO1 | 1.21343 | 2.466158 | 0.013657 |
| NOD2 | 1.300766 | 2.464926 | 0.013704 |
| C1QC | 1.109742 | 2.441898 | 0.01461 |
| BDKRB1 | 1.466645 | 2.434713 | 0.014904 |
| VSIG4 | 1.118909 | 2.404858 | 0.016179 |
| CXCL13 | 1.104253 | 2.343797 | 0.019089 |
| SRCIN1 | 0.782363 | -2.32877 | 0.019871 |
| CEACAM7 | 1.059732 | 2.298358 | 0.021541 |
| HMGA1 | 0.838551 | -2.26358 | 0.0236 |
| CPA1 | 1.580102 | 2.252962 | 0.024262 |
| SEMA6D | 1.240417 | 2.241385 | 0.025001 |
| LY86 | 1.194105 | 2.239835 | 0.025102 |
| PLAGL2 | 1.153721 | 2.235002 | 0.025417 |
| DERL3 | 1.205964 | 2.231362 | 0.025657 |
| S100B | 1.18991 | 2.229836 | 0.025758 |
| FOLR2 | 1.116804 | 2.220425 | 0.02639 |
| CD52 | 1.127686 | 2.219862 | 0.026428 |
| GSN | 1.149078 | 2.206078 | 0.027379 |
| GYPC | 1.182404 | 2.202399 | 0.027637 |
| TPSAB1 | 1.112715 | 2.190162 | 0.028512 |
| AIF1 | 1.134439 | 2.189095 | 0.02859 |
| MRC1 | 1.110237 | 2.179474 | 0.029296 |
| C1QA | 1.104921 | 2.172184 | 0.029842 |
| KIF12 | 0.90295 | -2.16018 | 0.030758 |
| TNFSF12 | 1.199998 | 2.158224 | 0.03091 |
| SLC22A23 | 1.265513 | 2.145318 | 0.031927 |
| FABP6 | 0.920784 | -2.1019 | 0.035562 |
| PLS3 | 1.167606 | 2.078734 | 0.037642 |
| ANTXR2 | 1.171856 | 2.047947 | 0.040565 |
| COL4A1 | 1.125148 | 2.041265 | 0.041225 |
| LPCAT3 | 1.293553 | 2.040869 | 0.041264 |
| TEF | 1.27001 | 2.038645 | 0.041486 |
| FAM107B | 1.156348 | 2.01639 | 0.043759 |
| GNL3 | 0.816248 | -2.01138 | 0.044285 |
| HAPLN3 | 1.118234 | 1.999181 | 0.045589 |
| PLCG2 | 1.257948 | 1.968827 | 0.048973 |
| NEK2 | 0.833373 | -1.9611 | 0.049867 |
| AP3M2 | 1.275418 | 1.960945 | 0.049885 |
| RTKN | 0.781629 | -1.9475 | 0.051475 |
| FAS | 1.170002 | 1.94295 | 0.052022 |
| IL1B | 1.079862 | 1.868919 | 0.061634 |
| CCL5 | 1.086819 | 1.86554 | 0.062106 |
| CTSW | 1.131561 | 1.849825 | 0.064339 |
| FBXL6 | 0.875991 | -1.83981 | 0.065797 |
| SIGLEC1 | 1.15783 | 1.821599 | 0.068516 |
| NAT10 | 0.807701 | -1.81282 | 0.06986 |
| PRMT3 | 0.828541 | -1.79876 | 0.072057 |
| ASGR1 | 0.863369 | -1.79284 | 0.072998 |
| ITM2C | 1.100978 | 1.789404 | 0.07355 |
| PNLIP | 1.409662 | 1.788838 | 0.073641 |
| FCGR3A | 1.073974 | 1.784821 | 0.07429 |
| GFUS | 0.866006 | -1.77563 | 0.075794 |
| CCNL2 | 0.862648 | -1.77245 | 0.076319 |
| PRR36 | 0.888454 | -1.75788 | 0.078769 |
| ATAD3C | 1.157823 | 1.75634 | 0.07903 |
| ZNF304 | 1.214088 | 1.742459 | 0.081428 |
| OR2I1P | 1.073182 | 1.73837 | 0.082146 |
| PDE8A | 1.254722 | 1.729128 | 0.083786 |
| PPP1R36 | 0.828374 | -1.70437 | 0.088311 |
| ADCY3 | 1.160313 | 1.694908 | 0.090093 |
| FAM111B | 0.873009 | -1.69298 | 0.09046 |
| BPIFB2 | 1.288605 | 1.67068 | 0.094785 |
| ALDH3B2 | 0.860249 | -1.66933 | 0.095052 |
| PUM3 | 0.83399 | -1.66249 | 0.096415 |
| ACACB | 1.221086 | 1.62579 | 0.103994 |
| SHROOM4 | 1.133981 | 1.621492 | 0.104912 |
| YAE1 | 0.882285 | -1.62005 | 0.105221 |
| TSHZ1 | 1.20052 | 1.617663 | 0.105735 |
| CDKN1A | 1.105657 | 1.615779 | 0.106142 |
| EEF1AKMT1 | 0.847227 | -1.60903 | 0.107611 |
| TRPM4 | 0.893467 | -1.6062 | 0.108229 |
| HM13 | 1.172568 | 1.592493 | 0.111274 |
| LTK | 1.105527 | 1.590752 | 0.111665 |
| QPRT | 0.94894 | -1.58325 | 0.113364 |
| LY6E | 0.935146 | -1.56004 | 0.11875 |
| ETV4 | 0.908703 | -1.5498 | 0.121189 |
| KLHL35 | 0.885342 | -1.54594 | 0.12212 |
| KIAA0513 | 1.176343 | 1.543174 | 0.122789 |
| C2CD4D | 0.822553 | -1.53828 | 0.12398 |
| FPGS | 0.839021 | -1.53093 | 0.125786 |
| POLR1G | 0.818508 | -1.52318 | 0.127715 |
| RNF182 | 1.138453 | 1.515283 | 0.129701 |
| TPM1 | 1.127239 | 1.512191 | 0.130485 |
| TOX | 1.097962 | 1.505405 | 0.13222 |
| NSDHL | 0.856244 | -1.49562 | 0.134752 |
| AKAP9 | 1.163141 | 1.490736 | 0.136031 |
| RUVBL2 | 0.868216 | -1.48866 | 0.136577 |
| H3C6 | 1.182798 | 1.486237 | 0.137217 |
| POLA1 | 1.200584 | 1.485708 | 0.137356 |
| GNL3L | 1.19747 | 1.485516 | 0.137407 |
| HOXB6 | 0.929101 | -1.47212 | 0.140989 |
| IL1RN | 1.065357 | 1.44722 | 0.147835 |
| BMP2 | 1.103887 | 1.447006 | 0.147895 |
| GMPR | 0.929991 | -1.43585 | 0.151045 |
| MACROD1 | 0.914598 | -1.40558 | 0.15985 |
| PAFAH1B3 | 0.893207 | -1.40209 | 0.160889 |
| ZMYND15 | 1.189708 | 1.396381 | 0.1626 |
| PHKA1 | 0.879853 | -1.39181 | 0.16398 |
| MUC4 | 1.063838 | 1.386509 | 0.165591 |
| CXCL8 | 1.042234 | 1.381231 | 0.167208 |
| TMEM132A | 1.118043 | 1.373323 | 0.169652 |
| FAM71E1 | 0.866433 | -1.35561 | 0.175224 |
| ITLN1 | 1.028042 | 1.350849 | 0.176744 |
| TOP1MT | 0.897661 | -1.32919 | 0.183786 |
| LXN | 1.100957 | 1.329066 | 0.183826 |
| P2RX4 | 1.17412 | 1.327307 | 0.184407 |
| HRCT1 | 0.935056 | -1.32645 | 0.184691 |
| LYG1 | 0.851592 | -1.32637 | 0.184718 |
| ZNF738 | 0.872239 | -1.27853 | 0.201063 |
| CLDN2 | 0.968471 | -1.2766 | 0.201742 |
| TTC9 | 0.906564 | -1.27616 | 0.201898 |
| TCOF1 | 0.883633 | -1.2683 | 0.204692 |
| PHYH | 0.904621 | -1.26585 | 0.205565 |
| DRD2 | 1.0816 | 1.264167 | 0.20617 |
| SRPX2 | 1.062387 | 1.263914 | 0.206261 |
| PPP1R13L | 0.90441 | -1.25478 | 0.20956 |
| TRIP6 | 0.939102 | -1.25451 | 0.209657 |
| CCL8 | 1.073304 | 1.253437 | 0.210047 |
| AGR3 | 0.962006 | -1.24872 | 0.211768 |
| TAZ | 0.86508 | -1.23468 | 0.21695 |
| HSF4 | 0.914349 | -1.23463 | 0.216967 |
| CLCA1 | 1.023335 | 1.231721 | 0.218053 |
| RAI2 | 1.075324 | 1.231059 | 0.218301 |
| EPDR1 | 1.065065 | 1.23075 | 0.218416 |
| CMSS1 | 0.862174 | -1.22851 | 0.219257 |
| NCR3LG1 | 1.090794 | 1.220419 | 0.222306 |
| TLCD3A | 1.114735 | 1.216599 | 0.223757 |
| SMPDL3A | 1.092124 | 1.186759 | 0.235323 |
| CELSR1 | 1.098151 | 1.181578 | 0.237373 |
| GUCA2B | 1.049837 | 1.180581 | 0.237769 |
| GSTP1 | 0.919675 | -1.17689 | 0.239239 |
| THUMPD2 | 0.853817 | -1.16793 | 0.242836 |
| CYP1A1 | 1.163295 | 1.153626 | 0.248653 |
| EXOSC4 | 0.920831 | -1.14949 | 0.250352 |
| COL7A1 | 1.067207 | 1.148238 | 0.25087 |
| TFAP4 | 0.861212 | -1.14636 | 0.251648 |
| SLC25A27 | 0.914558 | -1.13876 | 0.254802 |
| CDC20 | 0.910587 | -1.13389 | 0.256841 |
| ENOPH1 | 0.860959 | -1.1304 | 0.258309 |
| LIPG | 1.097469 | 1.129209 | 0.25881 |
| ZNF579 | 0.905917 | -1.11765 | 0.263717 |
| TRIM72 | 1.08309 | 1.113749 | 0.265387 |
| LRRC37A3 | 1.152271 | 1.106577 | 0.268477 |
| CCL24 | 1.043834 | 1.103934 | 0.269622 |
| CDC6 | 0.91753 | -1.09101 | 0.275266 |
| VEGFA | 1.104352 | 1.088309 | 0.276459 |
| RELL1 | 1.113057 | 1.087613 | 0.276766 |
| SDCBP2 | 1.05606 | 1.086397 | 0.277303 |
| MAT1A | 0.922511 | -1.08074 | 0.279814 |
| LDHD | 0.939751 | -1.07776 | 0.281139 |
| KANTR | 0.887017 | -1.07439 | 0.282649 |
| CENPF | 0.921913 | -1.074 | 0.282825 |
| IFRD2 | 0.899036 | -1.06995 | 0.28464 |
| REG4 | 1.020038 | 1.053289 | 0.292208 |
| ISYNA1 | 1.062354 | 1.052042 | 0.29278 |
| ADRB1 | 1.134643 | 1.04797 | 0.294652 |
| ABHD3 | 1.094602 | 1.028272 | 0.303822 |
| C9orf116 | 0.889685 | -1.02385 | 0.305906 |
| PTPRU | 1.053833 | 1.004629 | 0.315076 |
| PRR11 | 1.116568 | 0.995859 | 0.319319 |
| ETV5 | 0.933481 | -0.99203 | 0.321185 |
| COL9A3 | 0.961384 | -0.99157 | 0.321409 |
| MUC5AC | 1.037395 | 0.991315 | 0.321532 |
| PRSS56 | 0.945099 | -0.98081 | 0.326684 |
| EVX1 | 0.943275 | -0.97591 | 0.329107 |
| SPACA3 | 1.07062 | 0.968378 | 0.332856 |
| FEV | 1.256919 | 0.965624 | 0.334233 |
| MYO1A | 0.950595 | -0.95417 | 0.339998 |
| GLA | 1.119739 | 0.951794 | 0.341201 |
| TBC1D16 | 1.097396 | 0.949426 | 0.342404 |
| SERPINF2 | 1.058793 | 0.944117 | 0.34511 |
| TCEA3 | 0.948126 | -0.94211 | 0.346135 |
| GLTP | 0.875576 | -0.92743 | 0.353704 |
| TCF7 | 1.076926 | 0.925492 | 0.35471 |
| PLEKHB1 | 0.955629 | -0.91798 | 0.358629 |
| TMEM52 | 0.942308 | -0.91447 | 0.360469 |
| CEMIP | 1.047431 | 0.913937 | 0.36075 |
| PIEZO1 | 1.092687 | 0.913827 | 0.360808 |
| CDT1 | 0.934663 | -0.91227 | 0.361625 |
| PABPC1L | 0.955092 | -0.90309 | 0.366476 |
| MT1G | 1.031563 | 0.900299 | 0.367961 |
| OVGP1 | 0.91817 | -0.89832 | 0.369015 |
| ATIC | 0.902131 | -0.891 | 0.372929 |
| SLC1A1 | 1.057022 | 0.880122 | 0.378793 |
| ENGASE | 0.949766 | -0.87907 | 0.379366 |
| ASPHD1 | 1.056174 | 0.878469 | 0.379689 |
| PMEPA1 | 1.040483 | 0.870905 | 0.383806 |
| MOCS1 | 1.083527 | 0.870561 | 0.383994 |
| TEX43 | 0.934268 | -0.86651 | 0.386211 |
| PRMT5 | 1.087201 | 0.866064 | 0.386455 |
| UNC5CL | 1.05951 | 0.864867 | 0.387112 |
| EIF2S3 | 0.917496 | -0.85983 | 0.389881 |
| PRMT1 | 0.92865 | -0.8557 | 0.392164 |
| EVPL | 0.950175 | -0.84774 | 0.396581 |
| TINAG | 0.955315 | -0.84147 | 0.400087 |
| SDR16C5 | 1.034777 | 0.838111 | 0.401968 |
| SCML2 | 1.088497 | 0.82728 | 0.408078 |
| SIAE | 1.073236 | 0.823293 | 0.410341 |
| SULT1C3 | 1.079063 | 0.822649 | 0.410708 |
| IQGAP2 | 0.949204 | -0.82053 | 0.411912 |
| MCM8 | 1.109678 | 0.811971 | 0.416808 |
| BCL2L12 | 0.918881 | -0.80895 | 0.418546 |
| IRAK1 | 1.073883 | 0.808888 | 0.418579 |
| RAD51AP1 | 0.938776 | -0.8079 | 0.419147 |
| MT1H | 1.035914 | 0.806578 | 0.41991 |
| CCL28 | 1.040989 | 0.799522 | 0.423988 |
| PLCD3 | 1.058934 | 0.793088 | 0.427726 |
| SERPIND1 | 1.065749 | 0.79007 | 0.429487 |
| KLK5 | 0.894029 | -0.78874 | 0.430265 |
| BTBD16 | 0.897473 | -0.78755 | 0.430962 |
| RAE1 | 0.926621 | -0.78708 | 0.431233 |
| PDZK1 | 0.904007 | -0.78694 | 0.431316 |
| UTP14A | 0.931335 | -0.78611 | 0.431802 |
| RPS15 | 0.943887 | -0.78276 | 0.433768 |
| KIF15 | 0.929702 | -0.77465 | 0.438547 |
| NME2 | 1.037737 | 0.769763 | 0.44144 |
| KHDC4 | 0.936032 | -0.76014 | 0.44717 |
| VNN1 | 1.03245 | 0.756427 | 0.449393 |
| SPON1 | 1.030976 | 0.755112 | 0.450182 |
| H4-16 | 1.081401 | 0.754471 | 0.450566 |
| DPEP1 | 0.980212 | -0.75287 | 0.451528 |
| H2BC14 | 1.055843 | 0.744982 | 0.456282 |
| SERPINA7 | 1.067767 | 0.738381 | 0.460283 |
| ADGRG1 | 1.062685 | 0.73375 | 0.463101 |
| H2BC8 | 0.952503 | -0.73206 | 0.464133 |
| CDH3 | 0.949731 | -0.72935 | 0.465785 |
| RAC3 | 0.947858 | -0.72927 | 0.465838 |
| EREG | 1.025204 | 0.727774 | 0.466752 |
| CARS2 | 0.916258 | -0.72027 | 0.471357 |
| LGR4 | 1.063208 | 0.717023 | 0.47336 |
| H2BC17 | 1.047951 | 0.707887 | 0.479016 |
| FGFR4 | 0.963774 | -0.70431 | 0.481237 |
| DACH1 | 1.036956 | 0.703689 | 0.481627 |
| SPATA25 | 1.054634 | 0.69443 | 0.487413 |
| BST2 | 1.024843 | 0.694377 | 0.487446 |
| MEX3D | 1.059305 | 0.685914 | 0.492767 |
| PLXNA3 | 1.062983 | 0.685725 | 0.492886 |
| PIGR | 1.012924 | 0.685254 | 0.493184 |
| ACBD7 | 0.928642 | -0.68449 | 0.493663 |
| PODXL2 | 0.96343 | -0.66807 | 0.504089 |
| OCEL1 | 1.078737 | 0.666334 | 0.505198 |
| SLC17A4 | 0.945087 | -0.66509 | 0.505996 |
| ARMC2 | 0.927607 | -0.65627 | 0.511648 |
| ACAN | 1.074851 | 0.655573 | 0.512099 |
| ZNF514 | 0.925762 | -0.65189 | 0.51447 |
| NCAPD3 | 1.091745 | 0.640884 | 0.521598 |
| RPS6KA6 | 1.05999 | 0.639319 | 0.522615 |
| IL1A | 1.040997 | 0.638589 | 0.52309 |
| H3C2 | 1.035593 | 0.630237 | 0.52854 |
| MT1E | 1.020113 | 0.628081 | 0.529951 |
| TSPAN1 | 1.030925 | 0.626886 | 0.530734 |
| MEP1A | 1.021407 | 0.623816 | 0.532748 |
| COL27A1 | 0.954476 | -0.62108 | 0.534547 |
| PSPH | 0.948441 | -0.61331 | 0.53967 |
| SBSPON | 0.972834 | -0.60287 | 0.546598 |
| AGTRAP | 0.943995 | -0.59888 | 0.549253 |
| FMNL2 | 1.05981 | 0.595152 | 0.551742 |
| SOX9 | 0.956404 | -0.59304 | 0.553157 |
| WASHC2C | 1.072566 | 0.588068 | 0.556487 |
| GAS2 | 0.929768 | -0.58708 | 0.557152 |
| SYCP2 | 0.911918 | -0.58309 | 0.559832 |
| NEBL | 1.053644 | 0.580336 | 0.561688 |
| JADE3 | 1.056712 | 0.574697 | 0.565496 |
| BCAS4 | 1.055051 | 0.57203 | 0.567302 |
| HSD17B7 | 0.931281 | -0.57187 | 0.567411 |
| GPR143 | 0.972381 | -0.57161 | 0.567586 |
| HOXC6 | 1.04878 | 0.562946 | 0.573472 |
| DMRTA2 | 1.044748 | 0.559619 | 0.575739 |
| VMA21 | 0.940091 | -0.55352 | 0.579904 |
| ERCC6L | 0.9428 | -0.5527 | 0.580472 |
| TMEM160 | 1.025275 | 0.551314 | 0.581419 |
| MOCS3 | 1.052736 | 0.550647 | 0.581876 |
| LCN12 | 0.96009 | -0.54722 | 0.584231 |
| NKRF | 0.932007 | -0.5468 | 0.584516 |
| DAPL1 | 0.955752 | -0.54311 | 0.587052 |
| PTPRO | 0.97464 | -0.53941 | 0.589606 |
| CTTNBP2 | 1.038982 | 0.537591 | 0.59086 |
| CDK5RAP1 | 0.938704 | -0.53752 | 0.590906 |
| CCNP | 0.970293 | -0.53468 | 0.592869 |
| R3HDML | 0.963617 | -0.52687 | 0.598281 |
| QSOX2 | 1.067486 | 0.52366 | 0.600515 |
| THEM6 | 0.957825 | -0.5228 | 0.601112 |
| RNF125 | 1.047449 | 0.519438 | 0.603455 |
| MRPS2 | 0.955891 | -0.51936 | 0.603506 |
| ANKRD40CL | 1.03811 | 0.517848 | 0.604564 |
| GJC3 | 0.936886 | -0.50596 | 0.612888 |
| LRRC61 | 0.958639 | -0.49931 | 0.61756 |
| ARFGAP1 | 1.041907 | 0.492353 | 0.62247 |
| ZNF251 | 0.952671 | -0.48973 | 0.624328 |
| ZNF280C | 1.064976 | 0.486542 | 0.626583 |
| H2AC8 | 0.972379 | -0.48208 | 0.629749 |
| TRNP1 | 1.02145 | 0.480772 | 0.630679 |
| PKMYT1 | 0.961231 | -0.47842 | 0.632349 |
| PLCG1 | 1.03807 | 0.47721 | 0.633212 |
| GOLGA8B | 1.044614 | 0.474551 | 0.635107 |
| H4C4 | 1.027727 | 0.4725 | 0.63657 |
| ONECUT3 | 0.966934 | -0.46859 | 0.639366 |
| S100A3 | 0.968443 | -0.46456 | 0.642246 |
| DHPS | 0.94204 | -0.46456 | 0.642246 |
| ZFP3 | 1.053604 | 0.464314 | 0.642423 |
| KLHL17 | 0.958601 | -0.46147 | 0.644459 |
| CKB | 1.013274 | 0.457587 | 0.647249 |
| SPDL1 | 0.950459 | -0.45715 | 0.647564 |
| PAK6 | 0.950711 | -0.45433 | 0.64959 |
| FIBCD1 | 1.017608 | 0.45141 | 0.651694 |
| EIF2S2 | 1.047081 | 0.450102 | 0.652637 |
| B9D1 | 0.942516 | -0.44591 | 0.655659 |
| LIME1 | 0.955992 | -0.44212 | 0.658404 |
| AMELX | 0.953727 | -0.4392 | 0.66052 |
| RCBTB1 | 1.040907 | 0.438453 | 0.661058 |
| SELENOH | 1.029239 | 0.42876 | 0.668098 |
| LINC00514 | 0.966779 | -0.42207 | 0.672972 |
| SLC6A4 | 1.035637 | 0.418139 | 0.675846 |
| HK2 | 1.032481 | 0.414844 | 0.678256 |
| KRT15 | 1.040974 | 0.414287 | 0.678664 |
| C11orf86 | 1.019506 | 0.413384 | 0.679325 |
| XDH | 1.022432 | 0.410691 | 0.681299 |
| CROT | 1.045401 | 0.405419 | 0.68517 |
| TMEM255B | 1.036266 | 0.403361 | 0.686683 |
| H2AX | 0.966718 | -0.40333 | 0.686707 |
| CLIC3 | 1.01794 | 0.402291 | 0.68747 |
| IGFL2 | 1.024336 | 0.398603 | 0.690186 |
| ATAT1 | 0.958389 | -0.3979 | 0.690706 |
| SNTB1 | 0.972464 | -0.39648 | 0.691754 |
| SOCS2 | 1.044894 | 0.394048 | 0.693546 |
| TSPAN7 | 1.021893 | 0.390486 | 0.696178 |
| LCN2 | 0.989782 | -0.38606 | 0.69945 |
| RNF183 | 0.969798 | -0.38436 | 0.700714 |
| GABRE | 1.023818 | 0.37941 | 0.704384 |
| ANKZF1 | 0.952933 | -0.37908 | 0.704626 |
| ZDHHC9 | 1.034283 | 0.375143 | 0.707554 |
| AREG | 1.014473 | 0.373933 | 0.708454 |
| HSP90AB1 | 0.952305 | -0.37346 | 0.708805 |
| H2BC3 | 1.027186 | 0.368852 | 0.712238 |
| GJB3 | 0.982043 | -0.36681 | 0.713762 |
| YTHDF1 | 1.043378 | 0.366589 | 0.713925 |
| ASB9 | 1.022667 | 0.361829 | 0.71748 |
| ZBTB7C | 0.979306 | -0.35968 | 0.719085 |
| CACNA1D | 0.957006 | -0.3554 | 0.722289 |
| HAGHL | 1.025123 | 0.354838 | 0.722711 |
| TYRO3 | 1.036003 | 0.354415 | 0.723028 |
| CLCN4 | 0.970713 | -0.34736 | 0.728318 |
| F12 | 0.977605 | -0.34587 | 0.729441 |
| PKM | 0.967438 | -0.34173 | 0.732554 |
| TRIM16 | 0.967027 | -0.33571 | 0.737091 |
| H1-3 | 1.021966 | 0.332799 | 0.739286 |
| PDRG1 | 1.030311 | 0.331506 | 0.740262 |
| FIGNL1 | 0.968784 | -0.32688 | 0.743758 |
| PRDX4 | 1.029758 | 0.325808 | 0.74457 |
| POLD1 | 0.970219 | -0.32511 | 0.745101 |
| NT5DC2 | 0.973976 | -0.32186 | 0.747562 |
| ODAM | 1.015749 | 0.320766 | 0.748388 |
| MROH6 | 1.018069 | 0.319648 | 0.749235 |
| XPOT | 1.030068 | 0.318427 | 0.750161 |
| GNPDA1 | 1.037911 | 0.318058 | 0.750441 |
| SLC22A15 | 0.964701 | -0.3157 | 0.752227 |
| CYP39A1 | 0.978964 | -0.31208 | 0.754982 |
| BFSP1 | 0.964293 | -0.30871 | 0.757543 |
| CDCA7 | 0.980512 | -0.30723 | 0.758671 |
| PLK1 | 1.026937 | 0.303995 | 0.761132 |
| TMEM205 | 0.975962 | -0.30012 | 0.764083 |
| LRATD2 | 0.975677 | -0.29606 | 0.767182 |
| EIF4EBP3 | 1.018116 | 0.295412 | 0.767679 |
| C20orf27 | 1.021734 | 0.290063 | 0.771768 |
| NEIL3 | 0.966407 | -0.28696 | 0.774144 |
| LINGO1 | 1.023835 | 0.284652 | 0.775911 |
| TTLL3 | 0.968268 | -0.28433 | 0.776155 |
| SH2D2A | 0.973073 | -0.28381 | 0.776559 |
| NAT9 | 0.967425 | -0.2803 | 0.779246 |
| SLC35E4 | 1.021625 | 0.279609 | 0.779778 |
| LDLRAD3 | 1.02125 | 0.277323 | 0.781532 |
| TCFL5 | 0.97678 | -0.27661 | 0.782076 |
| H3C8 | 1.02382 | 0.267464 | 0.789112 |
| KLK12 | 1.012322 | 0.266916 | 0.789534 |
| GOLM1 | 0.981491 | -0.26269 | 0.792787 |
| TNNC2 | 0.992235 | -0.24865 | 0.803632 |
| LY6D | 1.017343 | 0.246625 | 0.805198 |
| TP53RK | 1.019886 | 0.240887 | 0.809643 |
| GDPD5 | 1.013996 | 0.232219 | 0.816368 |
| F7 | 1.020619 | 0.231303 | 0.817079 |
| MAOA | 0.988342 | -0.23061 | 0.817617 |
| SOX12 | 1.017852 | 0.228738 | 0.819073 |
| FARP1 | 1.019629 | 0.224539 | 0.822338 |
| SLC28A3 | 0.986493 | -0.22439 | 0.822451 |
| ANO9 | 0.98887 | -0.22368 | 0.823008 |
| UNCX | 0.638503 | -0.22311 | 0.823449 |
| LGALS4 | 0.98622 | -0.21807 | 0.827376 |
| GJB5 | 1.008434 | 0.209465 | 0.834085 |
| REG1A | 1.003436 | 0.201193 | 0.840547 |
| ZC3H8 | 0.974462 | -0.20118 | 0.840559 |
| USPL1 | 1.018347 | 0.19541 | 0.845072 |
| CGN | 1.016172 | 0.192863 | 0.847066 |
| PAPSS2 | 1.010341 | 0.192688 | 0.847204 |
| FBXO41 | 1.017474 | 0.191497 | 0.848136 |
| SLC5A6 | 1.01433 | 0.186812 | 0.851808 |
| POLQ | 0.976529 | -0.18386 | 0.854121 |
| NPTX2 | 1.005445 | 0.179077 | 0.857878 |
| MYEOV | 1.008458 | 0.17486 | 0.86119 |
| PCMTD2 | 1.012234 | 0.17359 | 0.862187 |
| PRKCG | 1.012706 | 0.173249 | 0.862456 |
| MAD2L2 | 0.981545 | -0.17075 | 0.864421 |
| ZNF572 | 0.98179 | -0.16891 | 0.865867 |
| SHLD1 | 0.986896 | -0.16452 | 0.869319 |
| COLEC10 | 0.976373 | -0.16417 | 0.869594 |
| GLOD5 | 0.986064 | -0.16135 | 0.871819 |
| RBBP8NL | 1.010739 | 0.152583 | 0.878727 |
| VDR | 0.98646 | -0.1473 | 0.882893 |
| H2BC6 | 1.01593 | 0.143857 | 0.885613 |
| TMEM63A | 0.988096 | -0.14255 | 0.886648 |
| H2BC7 | 1.012016 | 0.141142 | 0.887757 |
| TFCP2L1 | 1.007388 | 0.141058 | 0.887825 |
| UCKL1 | 0.989343 | -0.13689 | 0.891116 |
| ARRB1 | 1.014682 | 0.130636 | 0.896063 |
| GPRIN2 | 1.008337 | 0.119279 | 0.905055 |
| SCML1 | 1.008486 | 0.118423 | 0.905733 |
| H1-4 | 1.006854 | 0.11796 | 0.9061 |
| TJP3 | 0.991037 | -0.11473 | 0.90866 |
| H1-5 | 1.006648 | 0.114077 | 0.909177 |
| RLIM | 0.987994 | -0.11269 | 0.910276 |
| KCNH8 | 1.012111 | 0.108453 | 0.913636 |
| PCDHB8 | 0.987221 | -0.10813 | 0.913895 |
| CYP4F3 | 1.006332 | 0.105651 | 0.915859 |
| CCDC85B | 1.005548 | 0.104112 | 0.91708 |
| OGT | 1.007033 | 0.0925 | 0.926301 |
| BCL2L1 | 1.008226 | 0.090963 | 0.927522 |
| KLK10 | 0.996978 | -0.08824 | 0.929684 |
| KRT5 | 0.995523 | -0.08488 | 0.932357 |
| INHBB | 0.995684 | -0.08416 | 0.932931 |
| OBI1 | 0.992299 | -0.07871 | 0.937262 |
| IRX2 | 0.99614 | -0.07834 | 0.937559 |
| CDCA4 | 1.007565 | 0.076249 | 0.939221 |
| PTGR1 | 0.993023 | -0.07229 | 0.942368 |
| AMN | 0.996582 | -0.06858 | 0.945322 |
| STX16 | 0.9951 | -0.06592 | 0.947443 |
| FMO4 | 1.008371 | 0.065011 | 0.948165 |
| ST6GALNAC1 | 1.002168 | 0.05563 | 0.955636 |
| SLC6A6 | 0.99683 | -0.05559 | 0.95567 |
| CLCN5 | 0.994255 | -0.05367 | 0.9572 |
| PEX11A | 1.005444 | 0.046863 | 0.962622 |
| MTARC2 | 0.996325 | -0.04103 | 0.967269 |
| CYP2W1 | 0.999134 | -0.02992 | 0.976127 |
| RIPPLY3 | 0.997643 | -0.02607 | 0.979204 |
| WDR54 | 1.002471 | 0.025702 | 0.979495 |
| DMD | 1.002588 | 0.022302 | 0.982207 |
| MRPL12 | 1.001033 | 0.018917 | 0.984907 |
| DUSP2 | 1.001082 | 0.014738 | 0.988241 |
| TRMT6 | 1.001297 | 0.014299 | 0.988591 |
| PRR7 | 1.000365 | 0.006082 | 0.995147 |
| CD46 | 1.000449 | 0.005663 | 0.995482 |
| RCOR2 | 0.999673 | -0.00406 | 0.996761 |
| ARHGEF38 | 1.000309 | 0.002478 | 0.998023 |
| BTN1A1 | 1.000254 | 0.001844 | 0.998529 |

| gene | HR | z | pvalue |
| --- | --- | --- | --- |
| age | 0.934612 | -0.31432 | 0.075328 |
| gender | 1.060359 | 0.474682 | 0.063501 |
| stage | 0.741372 | -1.92606 | 0.00541 |
| T | 0.784551 | -0.9841 | 0.032507 |
| N | 0.889325 | -1.13492 | 0.025641 |
| M | 1.02899 | 0.146566 | 0.088347 |

Supplementary Table 2 Clinical Factors Associated with CRC Prognosis in Univariate Cox Analysis

Supplementary Figure 1


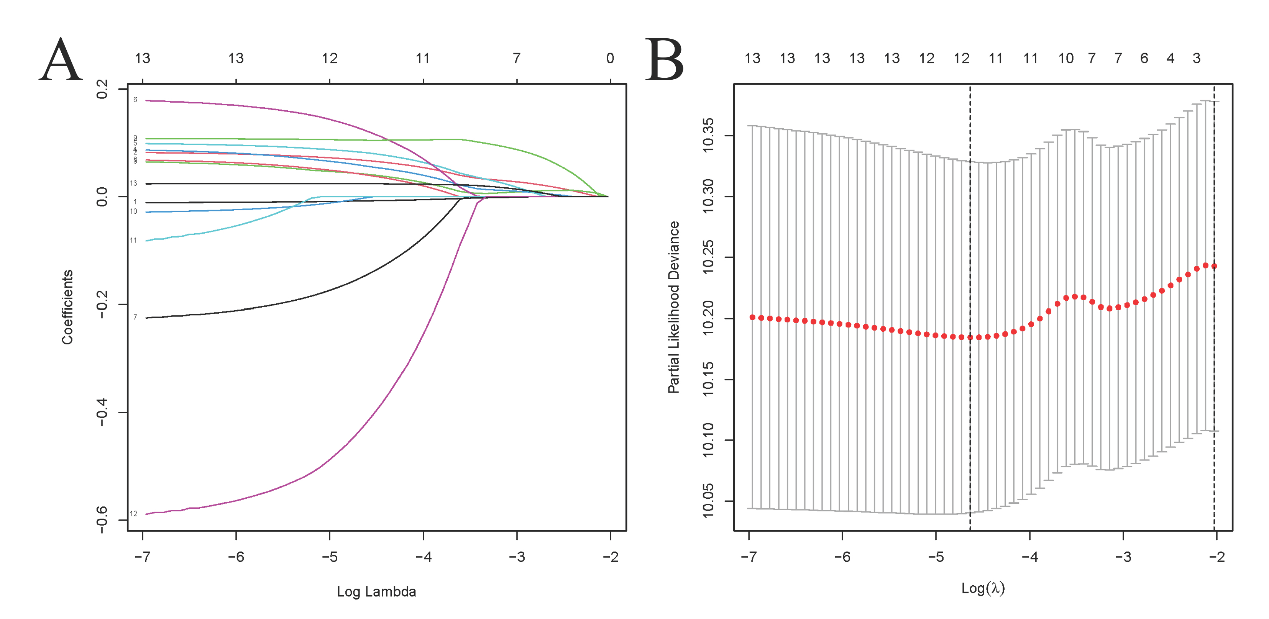


Supplementary Figure 2


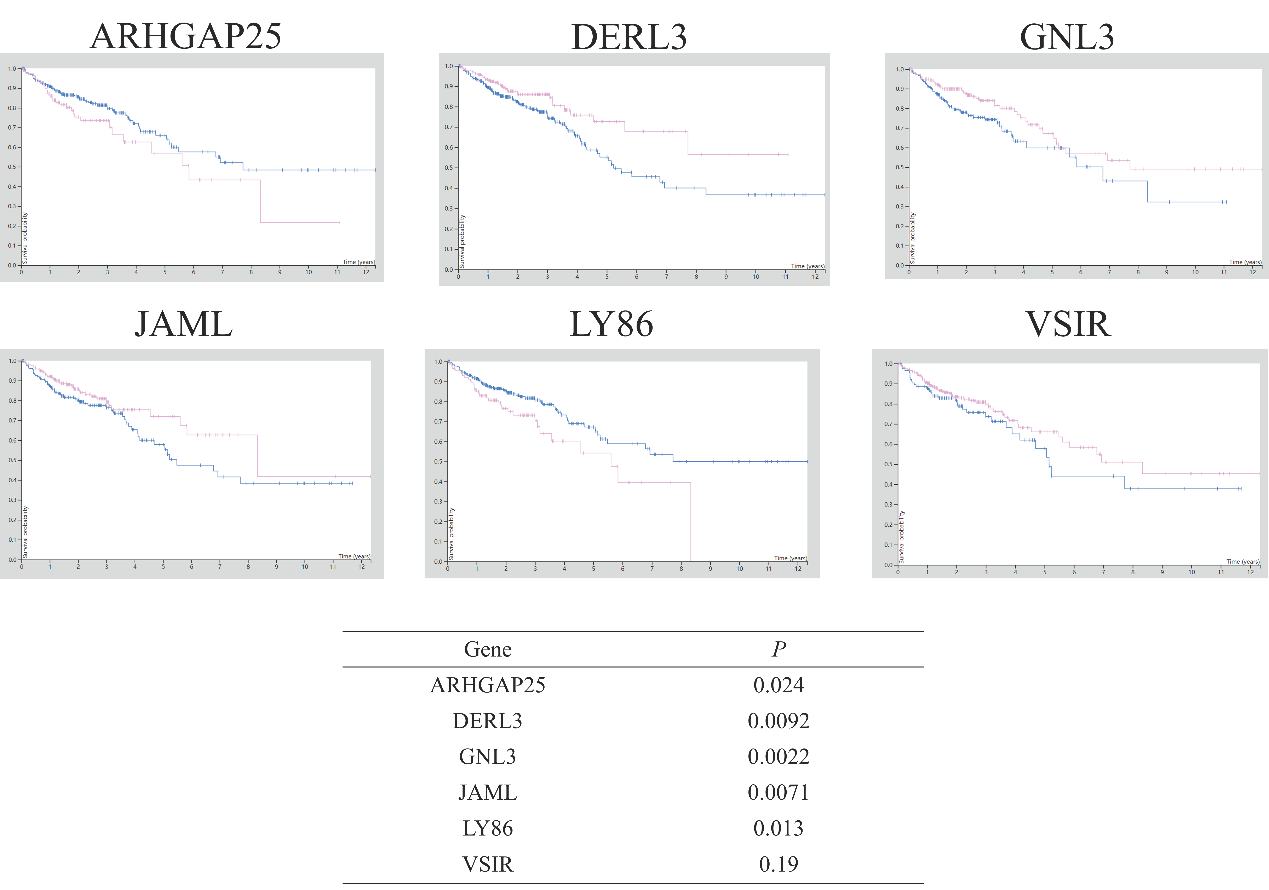

Supplement: Supplementary file 1 [file DataSheet_1.docx]
